# The Attitude of Great Writers towards Disease

**Published:** 1892-05-21

**Authors:** 


### The Attitude of Great Writers Towards Disease.

II.—SHAKESPEARE.

Dramatists are not, as a rule, much concerned about the springs which affect a man's bodily health. If he be sick, let him recover or die; neither the cause nor the cure of the malady is commonly of much importance to the action of the play; its unity would be disturbed by an analysis of either. It will, however, be readily observed that exceptions can occur to render the cause of a malady, as in poisons, or its cure, a matter upon which the whole plot may turn. This is distinctly the case in "All's Well that Ends Well," where the whole action depends upon the cure of the King by Helena with a prescription found among her father's papers, and upon the favour which is accorded to her in consequence.

The interest then which attaches to the plays of Shakespeare from a medical point of view does not by any means lie on the surface. There is, however, abundant evidence to show that Shakespeare was not only keenly observant of all that disturbs the balance of human health, but was also possessed by a discriminating appreciation of the efforts of those who endeavoured to restore it. This appreciation is nowhere more clearly shown than in the passage of Pericles where Cerimon, Lord of Athens, describes his enthusiasm for the science.

'Tis known, I ever
Have studied physic, through which secret art,
By turning o'er authorities, I have,
Together with my practice, made familiar
To me and to my aid the blest infusions
That dwell in vegetives, in metals, stones;
And I can speak of the disturbances
That nature works, and of her cures; which doth give me
A more content in course of true delight
Than to be thirsty after tottering honour,
Or tie my treasure up in silken bags
To please the fool and death.

Now the case for which Cerimon's skill is needed is remarkable. Queen Thaisa, having given birth to a child on board the ship which is bearing her and Pericles to Tyre, is presently seized with deadly faintness. This is mistaken by her attendants for death, and her body enclosed in a stately chest, is cast into the sea, and washed up shortly after on the shore of Ephesus. The restoration to life is described by Cerimon in words as full of accurate medical observation, as they are beautiful:—

Look how fresh she looks! They were too rough
That threw her in the sea. Make a fire within:
Fetch hither all my boxes in my closet. . . . .
Well said, well said; the fire and cloths,
The rough and woeful music that we have,
Cause it to sound, beseech you.
The viol once more: how thou stirr'st, thou block!
The music there!—I pray you, give her air.
Gentlemen,
This queen will live: nature awakes; a warmth
Breathes out of her: she hath not been entranced
Above five hours: see how she gins to blow
Into life's flower again.
* * * * *
She is alive; behold
Her eyelids, cases to those heavenly jewels
Which Pericles hath lost,
Begin to part their fringes of bright gold;
The diamonds of a most praised water
Do appear, to make the world twice rich.

It may be seen from this passage that the newest theories which advocate the introduction of music to the sick room, find support from Shakespeare. But Cerimon's use of music is full of deep significance. It is one expression among many of the vivid sense which Shakespeare held of the interdependence of mind and body. Not once or twice, but almost invariably, does he trace physical failure or suffering to this action of the mind upon the body. It is perhaps his recognition of the enormous difficulties of an art checked at every turn in the attempt to "minister to a mind diseased," which produced in him the reverent attitude which we have noticed towards it. A few passages must be quoted in illustration: Winter's Tale, Act 2, Scene 3—

*Leontes*: How does the boy?
*Servant*: He took good rest to-night,
'Tis hoped his sickness is discharged.
*Leontes*: To see his nobleness!
Conceiving the dishonour of his mother,
He straight declined, droop'd, took it deeply,
Fastened and fixed the shame on't in himself,
Threw off his spirit, his appetite, his sleep,
And downright languished.

See also 2nd Part of Henry IV., Act 4, Scene 4. Clarence speaks after the king swoons.

The incessant care and labour of his mind
Hath wrought the mure that should confine it in
So thin that life looks through and will break out.

The death scenes of Wolsey, Katherine, and King John suggest the same thoughts. Hear Nym and Pistol too (Henry V., Act 2, Scene 1) on the cause of Falstaff's illness.

*Nym*: The king hath run bad humours on the knight, that's the even of it.
*Pistol*: Nym thou hast spoke the right;
His heart is fracted and corroborate.

It is important to notice that in all these cases and in others which might be quoted, the bodily health is subdued not by any violent passion of grief, but by the crushing of the very spring of life within. It is shame rather than affliction which kills. Othello does not die of jealousy, nor Macbeth of remorse, nor any one, as Rosalind has taught us, of love. But it is the grinding shameful care which eats away a man's life.

And if the connection between body and soul is so close, we shall expect examples also of the revivifying effects of joy or indeed of any violent emotion. See Julius Cæsar, Act II., Scene 1.

*Ligarius*: Vouchsafe good morrow from a feeble tongue.
*Brutus*: O! what a time have you chose out, brave Caius,
To wear a kerchief. Would you were not sick.
*Ligarius*: I am not sick if Brutus have in hand
Any exploit worthy the name of honour.
*Brutus*: Such an exploit have I in hand, Ligarius,
Had you a healthful ear to hear of it.
*Ligarius*: By all the gods that Romans bow before,
I here discard my sickness. Soul of Rome!
* * * * *
Thou like an exorcist hast conjured up
My mortified spirit.

Compare also the noble Imogen in Cymbeline, Act III., Scene 6, fleeing to the Welsh mountains in disguise.

I should be sick
But that my resolution helps me.

And hear Menenius in his exaltation at being remembered by Coriolanus in the hour of his triumph. (Coriolanus, Act II., Scene 1.)

"A letter for me! it gives me an estate of seven years' health, in which time I will make a lip at the physician. The most sovereign prescription in Galen is but empiricutic, and to this preservative, of no better report than a horse drench."

It is clear from these very various examples that Shakespeare, with full appreciation of the science of medicine, is attracted principally to that mysterious point outside its realm where the forces of the soul meet either to contend or combine with those of the body. "What a piece of work is man; how noble in reason; how infinite in faculty!" He will not exalt the science at the expense of its "god-like" subject. Even in the moment of man's last and worst humiliation the dirge reminds us—

"The sceptre, learning, physic, must

All follow this, and come to dust."

